# Postoperative Pain Management among Surgically Treated Patients in an Ethiopian Hospital

**DOI:** 10.1371/journal.pone.0102835

**Published:** 2014-07-17

**Authors:** Tewodros Eyob Woldehaimanot, Tesfahun Chanie Eshetie, Mirkuzie Woldie Kerie

**Affiliations:** 1 Department of Pharmacy, Jimma University, Jimma, Ethiopia; 2 Department of Health Services Management, Jimma University, Jimma, Ethiopia; University of Michigan, United States of America

## Abstract

**Background:**

Incidence of postoperative pain has been reported to be between 47–100%. Ineffective postoperative pain management results in tangible and intangible costs. The purpose of this study was to assess the processes and outcomes of pain management in the surgical wards of Jimma University Specialized Hospital, Ethiopia.

**Methods and Findings:**

A prospective cross sectional study was conducted among 252 postoperative patients during February 13 to April 30, 2012. A contextually modified and validated (Cronbach’s α coefficient of 0.78) American Pain Society Patient Outcome Questionnaire was used to assess pain experience of patients. Patients’ charts were reviewed to assess the pattern of analgesic use. Incidence of postoperative pain was 91.4%, and remained high over 3 measurements (McNemar’s; p<0.05), and 80.1% of the patients were undertreated. The mean pain intensity, and pain interference on functional status were 6.72±1.44 and 5.61±1.13 on a 10 point Numerical rating scale respectively; both being strongly correlated(r = 0.86: p<0.001). Pain intensity was varied by ethnicity, education and preoperative information (ANOVA; P<0.05). Only 50% of the patients were adequately satisfied with their pain management. As needed (*prn*), solo analgesic, null analgesic, and intramuscular orders were noted for 31.3%, 89.29%, 9.7% and 20.1% of the prescription orders respectively. Though under dose, diclofenac and tramadol were the top prescribed medications, and only 57% of their dose was administered. Linear regression model showed that the predictors of satisfaction were sex of an individual and pain interference with functional status.

**Conclusion:**

Despite patients’ paradoxical high satisfaction with pain management, the majority of patients were inadequately and inappropriately treated. Thus, further research is needed to determine how best to break down current barriers to effective pain management.

## Introduction

It has been repeatedly confirmed by studies in the past 3 to 4 decades that 20 to 80% of patients undergoing surgery suffer from inadequately treated pain [1], [2] and pain is classified as a serious public health problem both in the developed [3] and in developing countries [4]–[6]. Despite this longstanding recognition of postoperative pain as a serious public health problem, and the increased knowledge and resources for treating pain, poorly controlled pain continues to pose a significant challenge to the management of patients in postoperative contexts [7]–[10].

In Africa, the issue of pain has been explored largely in relation to HIV/AIDS and cancer [11]–[13], even though pain from surgical procedures poses a far greater burden. A Human Rights Watch’s report showed that only 10% of these group patients are able to receive optimal pain management [14]. Even though various workshops and African Union summits adopted pain relief as basic human right [13], shortage of clinicians, stringent legal towards morphine access, and lack of knowledge left millions of people to suffer because of inadequate pain control [13], [15]. Ethiopia has almost nil morphine per capita which is on indicator of the equality of pain management [16].

In Ethiopia, a study conducted by the Ethiopian Public Health Association in 2005 showed that health care providers believes that pain was undertreated due to unstandardized practice, absence of medications and poor knowledge and attitude among professionals. The output of the survey was a step forward for the development of the 2007 National Pain Management Guideline [17]. The authors couldn’t find any study to demonstrate the quality of postoperative pain management at the level of patients.

The present study was conducted to assess the quality of postoperative pain management in the surgical wards of Jimma University Specialized Hospital (JUSH) by examining the incidence, intensity and interference of pain. Satisfaction and attitude of patients plus the patterns of pharmacologic and non-pharmacologic interventions with regard to pain management were also examined. After the conduct of this research, the department of Anesthesia of the University in conjunction with anesthesiologists from UK has taken a step to prepare a pain assessment tool and treatment guide for use by the surgical ward’s health care professionals.

## Materials and Methods

### Study setting and period

This study was conducted in the 3 surgical wards of JUSH during February 13 to April 30, 2012. The surgery department has 126 beds with a perceived 100% occupancy rate. Formal documentation of pain and preemptive analgesia are not common practices in this set up. Besides, anesthetists do not participate in the pain management of postoperative patients.

### Study design and Participants

**Figure 1:**
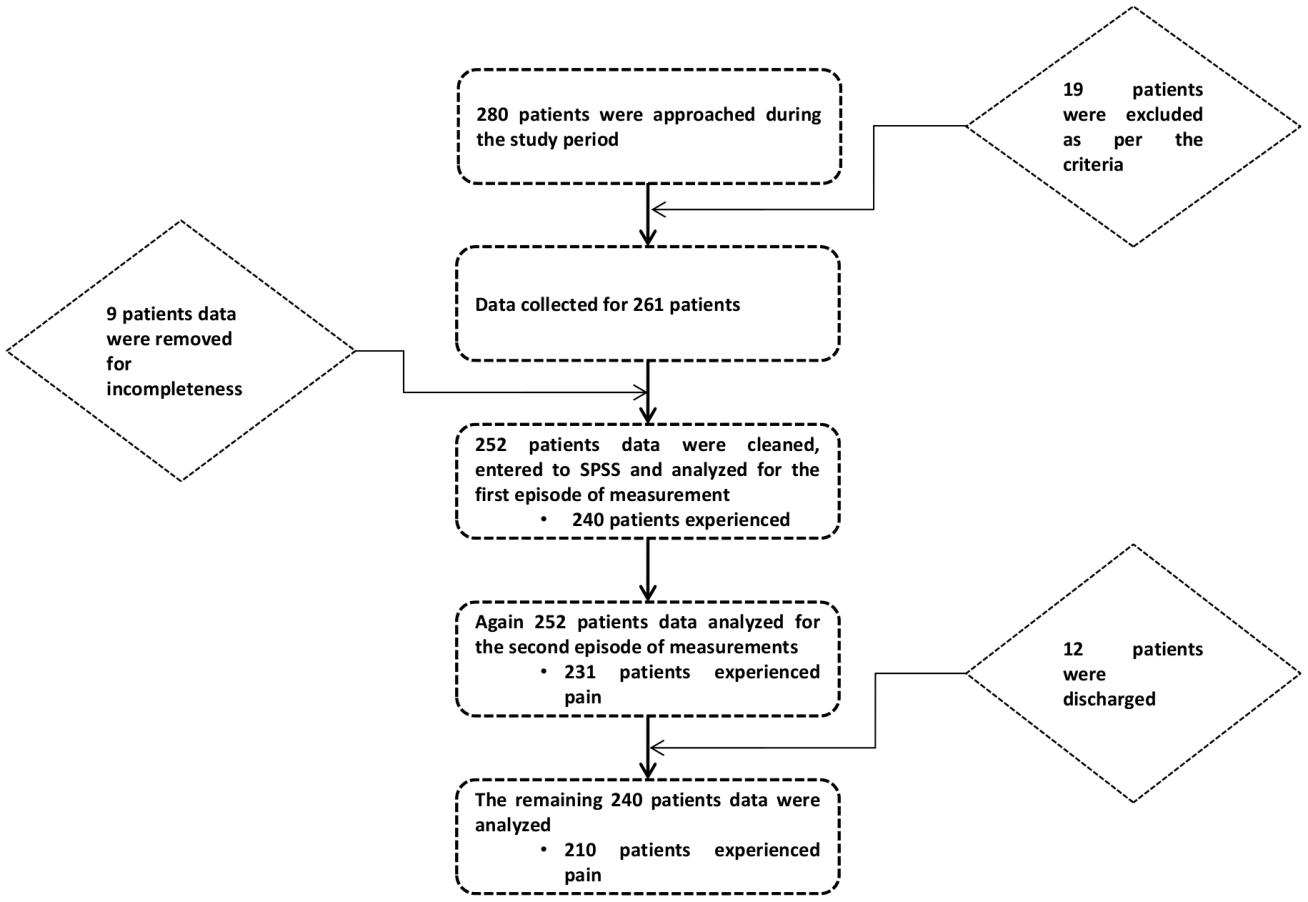
Schematic representation of patient recruitment and data analysis.

Prospective cross sectional study design was used to determine the quality of postoperative pain management. Consecutive, hospitalized patients aged over 18 years and within 24 and 72 h of surgery were invited to participate in the study. Of the 280 approached patients over the study period, 252 were recruited for analysis (Figure 1). Patients who had difficulty of communicating, unconscious and had documented psychiatric illness were excluded from the study.

**Figure 2:**
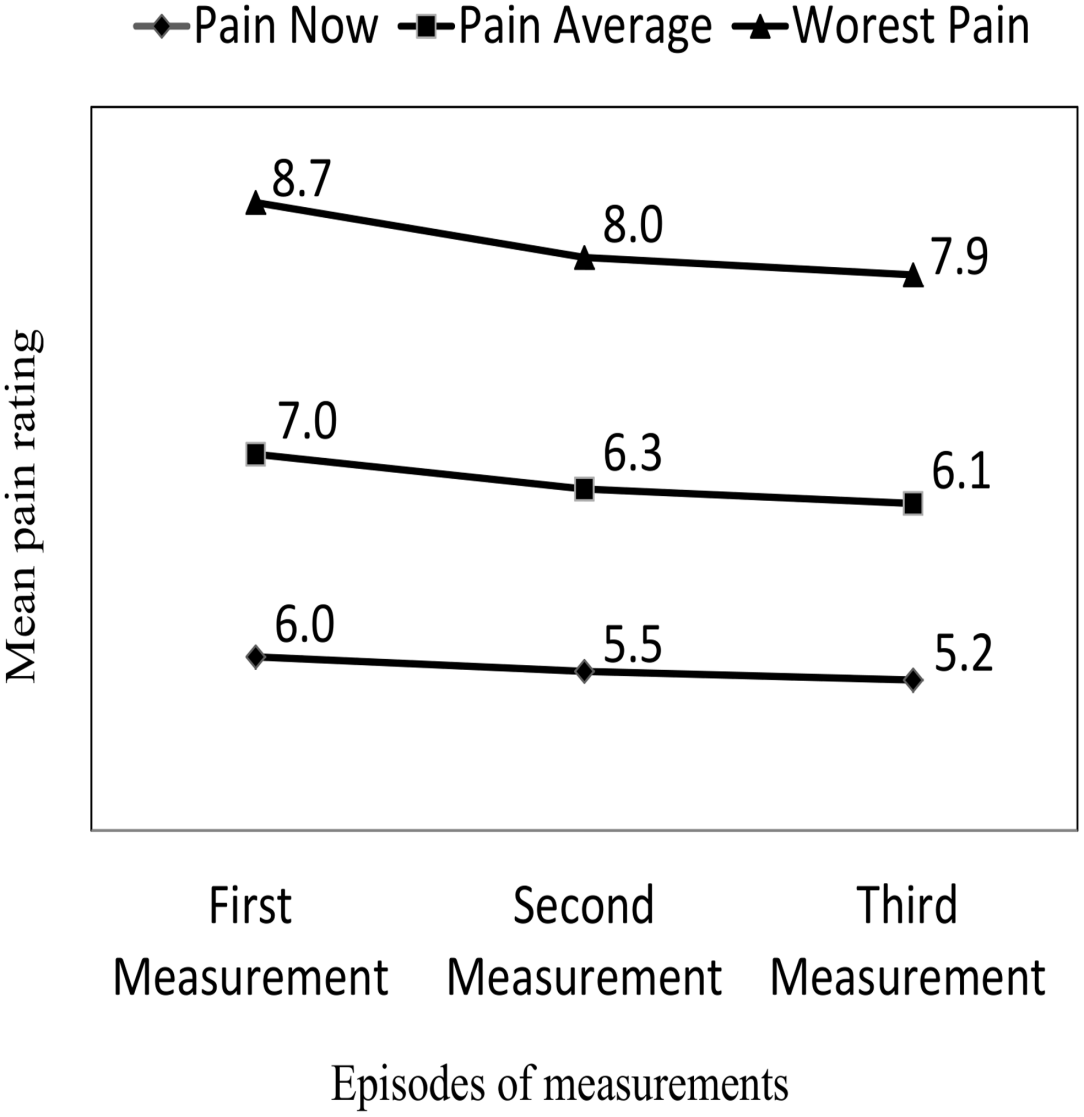
Mean pain intensity rating over the three measurements.

### Instruments and data collection methods

Based on the 1995 and 2010 versions of the American Pain Society Patient Outcome Questionnaire (APSPOQ), a contextually modified tool was prepared to collect data on patient satisfaction level, beliefs about pain and pain treatment, pain intensity, and effect of pain on function through face-to-face interview. These two tools are dependable and has been used extensively to survey pain in various contexts by multiple studies [4], [10], [18]. Both were designed by the American Pain Society (APS) and has incorporated a number of previously validated tools into its construction [19]. Over time and through its repeated usage and validation the APSPOQ has been translated into many languages other than English.

We predominantly used the 1995 version since it is extensively used and validated in both developed and developing countries. Initially three items, related to the use of non-pharmacological intervention and what perceived side effects patients come across, were picked from the 2010 version. After the pretest 2 items related to satisfaction with nurses’ and doctors’ were deleted since patients were not able to differentiate between a physician and a nurse. Similarly, the item that questioned patient’s request for medication change was removed, since it resulted in unanimously similar response of ‘No’. The item from the revised 2010 version of APSPOQ-R that assess the side effects of drugs was also erased because the side effects indicated were mainly related with strong opioids, which were not available in the study setting during the study period.

The final tool used in the current study had 13 items. The first item determines whether the patient experienced pain in the previous 24 hrs. The subsequent 3 items assess patients’ pain intensity level on the 0–10 Numerical Rating Scale (NRS). Item 5 relates to the degree to which pain interferes with six activities of daily living (general activity, walking, sleep, deep breathing and coughing, relationships with others and mood) on the same scale as previous. The next 2 items measures patients’ satisfaction with their overall pain management.

Then, 8^th^ item enquire patients’ alleged waiting time for analgesics when they ask for pain relief from 10 minutes or less to more than 60 minutes. If patients have pain at the time of interview, they would be asked whether they like something stronger for pain relief in item 9. In the next item patients were asked about their agreement (attitude and belief) levels to statements (patient barrier statements) related to pain and pain management on a 6 point scale of 0 (do not agree at all) to 6 (agree very much); higher score to these statements indicates higher levels of patients barriers to pain management.

The 11^th^ item relates to whether nurses or doctors inform patients about the importance of treating pain and reporting pain. The last two items assess patients experience with non-pharmacological management, and encouragement received from the health care professionals. Moreover, a structured data abstraction checklist was utilized to collect data on the pattern of pharmacological/non-pharmacological interventions and demographic characteristics of patients from the patient chart.

Those who were in a state to participate in the study were asked the first item. Patients with an experience of pain in the previous 24 hrs were interviewed with the APSPOQ in full, while patients with no experience in the previous 24 hrs were not asked the questions about pain intensity or the effect of pain on activities of daily living. Analgesics prescription and administration information for the previous 24 hrs were recorded for all participants by reviewing their medical records or interview of the patient and ward nurses. Satisfaction items were collected for the 1^st^ 24 hrs. Items on attitude and belief towards pain were collected for the 2^nd^ 24 hrs. While wait time, need for stronger dose, non-pharmacologic intervention were assessed for the 3^rd^ 24 hrs. The items on medication profiles, pain presence, pain severity and pain interference were interviewed for all of the 3 assessment episodes.

### Ethical statement

Permission to conduct this study was obtained from the **Institutional Review Board, Jimma University**. The data collectors (3 medical interns) first assessed patients’ ability to both comprehend the plain language information about the study and to participate in the study, by asking patients to repeat back the information provided. Patient’s written informed consent to participate in the study was obtained after comprehensive explanation of the purpose and procedure of the study. Patients were informed about their rights to refuse or withdraw, and about confidentiality of the individual information obtained. Additionally, any personal information was de-identified before the final analysis. During data collection process, data of patients who were at any risk of complication due to pain were shared with their medical and nursing team for intervention.

### Data analysis

Data were coded, cleaned and entered into and analyzed using the Statistical Package for the Social Sciences (SPSS Inc., Chicago, IL, USA) version 19.0. Descriptive statistics were computed to summarize the participants’ socio-demographic and clinical characteristics, patient satisfaction, and pain management processes (pharmacological and non-pharmacological interventions, patients request for pain relief and waiting time). The amount of analgesic patients received (mean dose ratio) was calculated as a proportion of dose administered to that of the total amount prescribed over the three episodes of measurements. The overall patients’ agreement levels to barrier statements related to pain and pain management were calculated as mean scores for each sub items. ANOVA, McNemar’s test, Pearson product moment were conducted to determine difference between groups, timed measurements, proportions, and link between variables accordingly.

The reliability of the APSPOQ used in this study was estimated by using the Cronbach’s α coefficient. Reliability estimates for the various subscales were 0.93 for pain intensity (3 items), 0.88 for pain interference (6 items), and 0.79 for beliefs about pain (7 items). The overall reliably for all items was 0.78.

Principal component analysis was used to reduce the 3 items of pain intensity, 6 items of level of pain interference on patients’ routines, and 7 items of barrier statements. The former two were measured over three days while the latter only at a point in time. The analysis was run independently for each the three factors. For the first two, the raw data matrix recoded so that the average of the three data point was taken during data reduction. However, the raw data matrix of the barrier statement was taken as it is since it was measured only once. Then, the appropriateness of the data was assessed using KMO’s and Bartllet’s test. The assessment of the Scree plot showed that only one component was enough for each category of measurement. Finally, the analysis for each was run again by setting the number of components to be one and the type of extraction Varimax. The generated scores were then used for subsequent analysis using ANOVA, correlations and linear regression.

To determine the adequacy of postoperative pain management Pain Management Index (PMI) was used. The PMI is based on a patient’s level of worst pain intensity and is categorized into 0 (no pain), 1 (1–3: mild pain), 2 (4–6: moderate pain), and 3 (7–10: severe pain) [20]. The pain score is then subtracted from the most potent level of analgesic drug therapies prescribed: 0 (no analgesic drug), 1 (non-opioids), 2 (weak opioids), and 3 (strong opioids) [21]. The index can range from –3 to +3. Negative scores indicate inadequate orders for analgesic drugs. Although the PMI was designed to evaluate the appropriateness of cancer pain management, several studies of postoperative pain management have established it as a useful indicator of adequacy in evaluating the range and appropriateness of pain treatments for hospitalized postoperative patients [22]–[24]. For all of the analysis a p-value of 0.05 was taken as a cutoff point for statistical significance. In the text, mean values with its corresponding standard deviation is expressed as (mean±standard deviation).

## Results

### Patient characteristics

Of the approached 280 patients 252 (90%) were included in the final analysis (); 162 (64.3%) were males. Patients were between 19 and 81 (40.4±15.5) years. Majority of the participants (71.4%) were Muslim by religion; and Oromo (72.6%) by ethnicity. Approximately 49% of the patients had no any formal education. Only 9.5% of the patients demonstrated previous surgical history of any type. The patients stayed in the wards for 0 to 28 days (5.2±5.1) prior to their surgery. Surgical interventions were categorized according to approach, with the largest being abdominal 90 (35.7%). The majority, 228 (90.5%), received general anesthesia, while the remaining subjects received spinal anesthesia. A large proportion of subjects, 198 (78.6%), had scheduled surgical interventions. The mean duration of all the surgical procedures was 82.9±43.7 minute (Table 1).

**Table 1:** Demographic and clinical characteristics of participants (n = 252).

Variables	N (%)
**Sex**	
** **Male	162 (64.3)
** **Female	90 (35.7)
**Religion**	
** **Muslim	180 (71.4)
** **Christian	48 (19.1)
** **Other*	24 (9.5)
**Ethnic** **origin**	
** **Oromo	183 (72.6)
** **Southern Ethiopia	42 (16.7)
** **Others**	27 (10.7)
**Educational** **status**	
** **Higher Education	18 (7.1)
** **High School	36 (14.3)
** **Elementary	54 (21.4)
** **Basic	21 (8.3)
** **Illiterate	123 (48.8)
**Previous** **surgical** **history**	
** **No	228 (90.5)
** **Yes	24 (9.5)
**Surgical** **Procedures**	
** **Urological	66 (26.2)
** **Abdominal	90 (35.7)
** **Endocrine	15 (6.0)
** **Orthopedics	33 (13.1)
** **Thoracic	6 (2.4)
** **Skin, Muscle& Soft Tissue	42 (16.7)
**Category** **of** **surgery**	
** **Elective	198 (78.6)
** **Emergency	54 (21.4)
**Anesthesia** **type**	
** **General	228 (90.5)
** **Spinal	24 (9.5)

*Includes: “Waqafata”; those without religion affiliations.

**includes: Tigrai, Amhara, Gambela.

### Principal component analysis

The principal component analysis generated one component for each group of items. The variations explained by the generated scores were 77%, 75% and 66% for pain intensity, interference and belief items respectively. The pain intensity component has the strongest correlation with the average pain item (r = 0.91), the interference one with mood (r = 0.87), while the barrier (belief) component with the addiction statement (r = 0.82).

### Incidence and severity of pain

When interviewed 240 (95.2%), 231 (91.7%), 210 (87.5%) of the patients had experienced pain in the 1^st^, 2^nd^, and 3^rd^ previous 24 hrs respectively. The pain incidence of the first assessment was significantly higher than that of the second day (McNemar’s; p = <0.05). The mean pain intensity for the three consecutive episodes were: (5.56±1.76), (6.46±1.33) and (8.16±1.23) for pain now, pain average, and pain worst pain respectively. A consistent and significant (*P*<.001) decrease in pain intensity was observed over time ().

Barrier to pain management (r = −0.17: p<0.05), age (r = −0.20: p<0.001) and duration of surgery (r = −0.16: p<0.05) were negatively and mildly correlated with pain intensity. Even though, it was not statistically significant, the ratio of drug administered and longer ward stay showed an inverse relation with pain intensity. In as much as the previous, patients who rated higher pain intensity were from the southern region of the country (P<0.05), more educated (P<0.05), and more pre-informed (p<0.05).

### Pain Interference with functional status

Mean of pain interference over the 3 days in decreasing order were: walking ability (6.77±1.44), general activity (6.57±0.98), mood (5.83±0.97), sleep (5.59±1.22), coughing and deep breathing (4.89±1.19), and relation with others (4.01±1.02). The overall mean over the 3 days was 5.61±1.13, and it decreased significantly over time (p<0.001). Higher interferences were reported by individuals who; rated higher pain (r = 0.86: p<0.001), spent shorter duration in surgical procedures, were younger, exhibited less hindering attitude, and were more educated (p<0.05).

### Attitude and belief (Indicators of barriers to adequate pain management from the patient side)

Patients were asked to rate on a 0 to 5 scale (0: do not agree at all, 5: agree very much) how much they agree with seven statements focused on common pain management misconceptions. The higher the rate on the likert scale, the greater the barrier (hindering attitude) to adequate pain management. Patients reported relatively higher levels of agreement with all 7 statements, with the highest level of agreement for the statement on addiction (3.8±1.22) (Table 2).

**Table 2:** Patients’ agreement with barrier statements, sorted in increasing order.

Statements	Mean ± SD
Pain medication should be ‘saved’ in case the pain gets worse	2.3±1.0
Complaints of pain could distract the doctor from treating my underlying illness	2.4±0.9
Good patients avoid talking about pain	2.5±0.8
It is easier to put up with pain than with the side effects that come with pain treatments	2.8±1.4
Pain medication cannot really control pain	3.3±1.1
The experience of pain is sign that the illness has gotten worse	3.5±1.5
People get addicted to pain medication very easily	3.8±1.2

### Process of postoperative pain management and adequacy of management

Only few patients (2.5%) reported that they received pain medication within 15 minutes of complain of pain. However, a large number of patients (70.8%) never asked for pain medication during hospitalization. Despite the fact that only few patients requested for pain medication, 42.2% of the patients responded “Yes” when they were asked whether they want stronger medication for pain relief. Moreover, 14.2% of the patients reported that they never received the analgesics they requested (Table 3). Most patients, 91.2% (n = 230) reported that their physicians or nurses haven’t discussed with them the importance of pain management.

**Table 3:** Processes of pain management.

Variables	N (%)
**Waiting Time after requesting analgesics (n = 252)**	
** **Below 15 minute	6(2.5)
** **Up to 30 minute	4(1.7)
** **Up to 1 hour	22(9.2)
** **Beyond 1 hour	4(1.7)
** **Asked, Never received	34(14.2)
** **Never asked	170(70.8)
**Want stronger dose of medication (n = 252)**	
** **No	133(57.8)
** **Yes	97(42.2)
**Received pre-information (n = 252)**	
** **No	230(91.3)
** **Yes	22(8.7)
**Help to use non-pharmacological ways (n = 252)**	
** **Never	200(83.3)
** **Sometimes	31(12.9)
** **Often	9(3.8)
**Name of prescribed medications (n = 279** * **)**	
** **None	27 (9.7)
** **Diclofenac	111 (39.8)
** **Tramadol	120 (43)
** **Pethidine	3 (1.1)
** **Unspecified	18 (6.5)
**Route of administrations (n = 234** § **)**	
** **IV	186 (79.5)
** **IM	48 (20.5)
**Prescriber’s qualifications (n = 244** † **)**	
** **Specialist	42 (16.7)
** **Residents	202 (80.2)
** **Unspecified	8 (3.2)
**Frequency of medications (n = 234** § **)**	
** **BID	43 (17.1)
*** *** *prn*	79 (31.3)
** **QID	40 (15.9)
** **TID	90 (35.7)

*N is 279, because 27 patients received two drugs.

§ 234 is the orders with medication regimen written.

† 244 is patient cards with physician qualification written on.

Even though the dominant pattern of analgesic prescriptions found in this study was scheduled one (68.7%); as needed (i.e., *prn*) orders for analgesics were noted in 31.1% of the orders. Analgesics that were prescribed with a fixed interval were administered 54% of the time; however, *prn* orders, irrespective of analgesic category, were only administered 5% of the time. The majority of patients were prescribed solo analgesics (89.29%). The remaining (10.71%) were prescribed dual analgesic: namely Diclofenac and Tramadol. It was also observed that forty five patients (10.1%) did not have prescription for any kind of specific analgesic. On average, the dose administered for Diclofenac was only 56.2% of the prescribed while for Tramadol it was 57.9% (Table 3).

The mean total daily dose (in mg) administered over the three measurements episodes were: 122.5±21.3, 88.8±14.2, 81.6±12.7 for Tramadol, and 101.4±19.3, 81.1±11.8, 64.9±9.5 for Diclofenac. The dose of the drugs administered decreased consistently and significantly over time (*P* = .0001). Change of prescription of pain medication was reported only in 3% of the cases. The most frequent non-pharmacological ways of managing pain were tolerating pain (84.4%), changing position (83.7%), and having family support (81.9%). Approximately, 83% of the participants did not receive support from the health care providers in this regard.

As PMI is a novel method for evaluating the range and appropriateness of pain treatments in term of prescription. It is used to determine adequacy of treatment. One hundred and ninety four participants (80.1%) received ineffective pain medication. The remaining 48 patients (19.9%) received adequate to good pain medication.

### Satisfaction with pain management

In response to questions about overall satisfaction with management of pain, 117 (50%) patients were satisfied or very satisfied. With a range of 1–6, the mean overall satisfaction with pain relief management was 4.22±1.51 for all patients. Though it was weak the only variable correlated with satisfaction level was pain interference (r = −0.16, p<0.05). Significant differences were between male and female (P = 0.04); female being less satisfied.

The only two variables shown to be associated with satisfaction and verified to be predictors of satisfaction (F (2,225) = 5.311, p = 0.006, adjusted R^2^ = 0.16) were sex of an individual and interference of pain with functional status. It was found that a unit increase in pain interference on functional status results in a 0.167 unit decrease in satisfaction level with pain management (B = −0167, 95%C:−0.852, −0.028). Similarly, females’ satisfaction with pain management is lower than that of male by 0.137 (B = −0.137, 95%CI: −0.439, −0.057) (Table 4).

**Table 4:** Regression model for patient characteristics and clinical outcomes as predictors of satisfaction.

	Coefficients				
Variables	Unstandardized		Standardized	95% CI for B	
	B	SE	B	Lower Bound	Upper Bound
Constant	4.802	0.297		4.218	5.386
Interference of pain	−0.248	0.097	−0.167	−0.852	−0.028
Sex	−0.44	0.209	−0.137	−0.439	−0.057

## Discussion

This study is the first to evaluate the quality of postoperative pain management in JUSH using the quality improvement standards recommended by APS [25]. In addition, it used multiple time measurements of the incidence, intensity and interference of pain, and the pattern of pharmacologic and non-pharmacologic interventions. Such kind of study does not require the same evidentiary and rigorous populations as is usually demanded of more general research as it is not primarily intended to generate new knowledge of widely generalizable or universal value [26]. Thus, the generated data could reliably show points of interventions to improve the quality of postoperative pain management and serve as a baseline for continuous audits to come.

Findings from this study should be interpreted and understood within the context of barriers of pain management in Africa, and postoperative pain management in Ethiopia, where, at the time of data collection, there was one national general guideline prepared by the Federal Ministry of Health (MOH) [17]. This guideline is expected to chaperon professionals to effectively manage postoperative pain. This guideline was prepared to be in tune with the World Health Organization (WHO) ladder of pain management.

In hospitals of most developing countries (Nigeria, Kenya, Uganda, South Africa, China, Columbia and Malaysia) pain management is derived from the medical staff’s experience, and is not always consistent with recommendations from organizations such as the APS [11], [12], [15], [27]–[30]. Postoperative pain medications are still prescribed on an as-needed basis, requiring patients to request pain medication, and interventions are implemented when patients are in severe pain [15]. In most acute care settings, pethidine and intramuscular injection are the commonest prescription orders, neither of which is recommended by pain management guidelines [25], [29]. Maximum doses of paracetamol and non-steroidal anti-inflammatory drugs are rarely used unlike in the developed world [2], [28]–[31]. The high cost of opioids in developing countries compounded this problem. The above mentioned challenges for better pain relief are also observed in our study.

The incidence and level of severity of pain reported in this study is higher than those from most Western and developing countries [2], [10], [22], [24], [32]–[34]. But, an earlier study done in China reported a 100% postoperative pain incidence [35]. Although, as expected, all pain intensity scores decreased over time, patients in this study continued to have an average pain score of greater than 6, and 79% had a worst pain score of greater than 6 on the third postoperative day. Besides, 34% of patients responded that they required stronger pain medications in the third postoperative day. Such troublesome level of postoperative pain may be explained by the high negative PMI scores and absence of strong opioids. Similarly, the inappropriate prescription and administration of analgesics plus only surgeon based care augment this too. Postoperative care delivered by multidisciplinary team including anesthesiologists/anesthetists in Acute Pain Service setups has shown a better outcome [36], [37].

Age, attitude, information status and education are often considered indicators of the patient’s perception of power [10], [33]. In our study, less pain intensity was reported by the elderly and patients with poor attitude, and less educated and informed. This finding underpins that fact that these groups of patients are highly vulnerable and therefore needs greater attention. Similarly, increased duration of surgery was negatively correlated with pain intensity. This may be due to the fact that these groups of patients may receive higher amount of anesthesia, which may decrease pain perception.

The role of ethnic background (ethnic origin) to affect pain perception and satisfaction was mentioned in studies done in Singapore and Nigeria [15], [38], [39]. In our study, individuals from the southern part of the country were found to rate pain higher. This finding cannot be explained with the information at hand, thus we recommend further investigation to explore the matter. The fact that the amount of drug administered was found to affect pain intensity likely demonstrates the amount of drug prescribed was suboptimal from the very beginning.

The interference scores reported by the patients in this study were higher than those reported in other studies with more heterogeneous samples of hospitalized patients China, USA, and South America [32]–[34]. As with the case of pain intensity, mean pain interference scores also decreased over time. It was well correlated with pain intensity scores. Taking this correlation in to account, investigators of this study also came to the conclusion made by other studies that pain interference scores to be used as monitoring indicator of the quality of postoperative pain management [10], [32]. However, future studies are non-optional to determine what specific pharmacologic and non-pharmacologic interventions can be implemented in decreasing pain intensity scores and its interferences.

The majority of patients never requested pain medication or changes in pain medication. In comparison to patients from other countries [33], [34], [40], the data showed that patients of the current setting were less likely to request pain medication even if they were suffering high levels of pain. This result, being harmonious with high level of barrier, may imply that patients were more passive in pain management or health care in general, and are less likely to verbalize their needs and concerns. A South African study conducted among 45 chronic patients showed that pain interference with quality of life was high among uninformed patients [11]. Due attention must be taken by the health care team in keeping patients well informed about the importance of pain relief.

Regarding adequacy of pain management, 80.1% of the population was poorly managed in the current study site. For comparison, 60.2% were inadequately treated for pain in Chinese population [34] whereas only 36% had inadequate treatment in a medical surgical sample in the United States [22]. In a secondary analysis of a large postoperative sample of the same study in United States after 3 years, reported an overall 30% rate of under treatment [41]. The negative PMI score represents a gross and minimum estimate of inadequate postoperative pain management. It would be reasonable to estimate that a mere presence and prescription of strong opioids would have minimized the negative PMI significantly.

The principal means the study participants used to cope with pain were tolerating pain, self-prayer, family help, and changing positions. This finding is consistent with findings from Chinese and Mexican population [24], [34]. However the use of music, guided imagery, prayer by others, and other sophisticated methods which are commonly used by USA, Hispanic and Canadian samples are not available in our sample [22], [32], [42]. That fact that tolerating pain is opted for by our patients goes well with finding most agreed high with the barrier statements.

A paradoxical high satisfaction despite high pain intensity was observed in our study samples. But, the overall satisfaction of patients was lower than reported by most studies in developed and developing countries [2], [8], [32]. The main reasons identified by most studies for higher rate of satisfaction despite the presence of high pain intensity are the exceptionally good caring attitude of health care professional, presence of frequent pain assessment, high rate of preoperative pain education, and presence of good communication environment [10], [32], [40], [43]. In our study, the majority of participants reported they had not received pain management education, and backing on the use of non-pharmacological methods was minimal. Similarly, the reason mentioned for dissatisfaction were overlooking of the clinician to patients’ requests. All these together indicate low level of communication to patients which might explain the relatively lower satisfaction rate.

The correlates of satisfaction in our study were gender and interference of pain. Female’s higher intensity of pain and dissatisfaction were also demonstrated by studies in Western countries [43]–[45] and eastern countries [33], [35]. A south African study also reported this similar scenario [11]. The commonly proposed reason for this is that females are more socially acceptable to express pain and dissatisfaction [46], [47], but it is yet to be determined as further studies are to be done in this area. Even though, expectation of pain and the perceived degree of pain relief were the most common predictors of satisfaction reported [26], [48], [49], gender and interference of pain were also reported in few instances [24], [26]. Taken together, both streams of evidences well support that appropriate relief of pain with appropriate medications could bring optimal satisfaction.

Although this study is the first to use the APSPOQ, in Ethiopia, to evaluate the quality of postoperative pain management in this setup, limitations that can affect generalizability need to be noted. First, collection of data in a single site and from patients operated by a limited number of surgeons presents a limitation to the external generalizability of this study. Second, the majority of patients were male which might decrease pain intensity and interference ratings, because women are more likely to report higher pain and interference scores than men. Thirdly, the majority of the patients had similar socio-demographic characteristics which also affect variability of responses. Furthermore, there are known multifactorial influences related to setting of facility, patient’s expectations, professionals’ attitude and knowledge on pain experience that have not been explored in this study.

In conclusion, postoperative pain management was suboptimal among postoperative patients of the surgical wards in JUSH. This was evidenced by the high incidence of postoperative pain and its consequences. Unstandardized treatment use, poor patient attitude, shortage of pain clinicians, lack of strong opioids, and lack of knowledge might be background causes. Thus, further research is needed to determine how best to break down current barriers to effective pain management.
